# Lipase-catalysed changes in essential oils revealed by comprehensive two-dimensional gas chromatography

**DOI:** 10.1007/s00216-023-04729-0

**Published:** 2023-05-15

**Authors:** Michelle S. S. Amaral, Milton T. W. Hearn, Philip J. Marriott

**Affiliations:** Australian Centre for Research On Separation Science, School of Chemistry, Monash University, Wellington Road, Clayton, VIC 3800 Australia

**Keywords:** Enzymes, GC×GC‒MS, Biocatalysis, Esterification, Natural products

## Abstract

*Candida antarctica* lipase A (CALA) was applied for the chemo-selective enzymatic transesterification of terpene and phenyl alcohols in 35 different essential oil samples. Comprehensive two-dimensional gas chromatography with mass spectrometry (GC×GC‒MS) analysis enabled the separation and tentative identification of a cohort of 125 compounds, allowing the instant visualisation of the reaction process changes, amid the complex chemical background of the samples. The results indicate that 42 out of 79 alcohols so-identified were fully or partially esterified within 48 h of reaction, with primary alcohols being the substrates of preference of the enzyme (90–100% conversion), followed by secondary alcohols (mostly ~ 80–100% conversion). No significant conversion of tertiary alcohols and phenols was observed using the tested conditions. Overall, the enzyme’s performance was consistent for primary alcohol substrates identified in multiple samples of different compositions. The observed selectivity, efficiency, robustness, scalability (enzyme/substrate working concentration ratio > 1:160), potential reusability, mild reaction conditions, and other factors make this process a greener and more sustainable alternative for industry applications, particularly for the manufacture of novel flavours and fragrances.

**Graphical Abstract:**

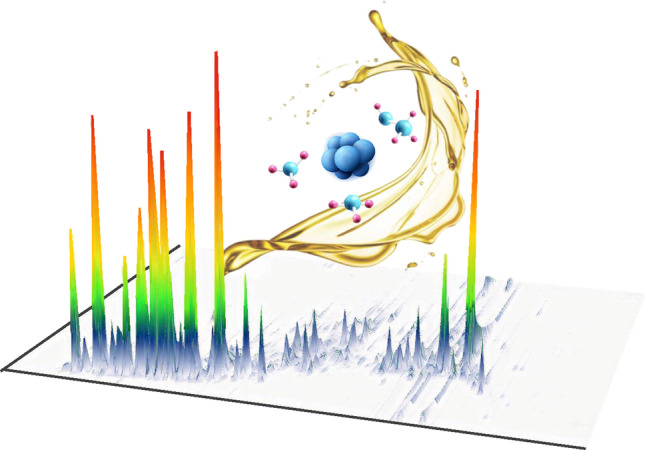

**Supplementary Information:**

The online version contains supplementary material available at 10.1007/s00216-023-04729-0.

## Introduction

In the past two decades, green and biotechnological processes have quickly evolved and many have been adopted in different industrial applications. The use of enzymes as biocatalysts for the synthesis of fine chemicals, such as flavours and fragrances (F&F), is an increasing trend in the market. This processing option has potential to produce high-value compounds by the conversion of abundant, less expensive, and renewable raw materials, and using a more chemically and energy efficient, chemo-, regio-, and stereo-selective, reusable, biodegradable, and natural catalyst. It can also improve the safety, sensory quality, and biological activity of F&F products, while keeping their “naturally derived” labelling. These appealing advantages, as opposed to the often harsher synthetic chemical processes or extraction/isolation methods from scarce natural sources, are some of the reasons for the growing adoption of biocatalysis [1–5].

In this context, hydrolytic enzymes, such as lipases, have been established and increasingly dominate the industrial enzyme market. Versatile and robust, lipases essentially act on carboxylic groups, being able to catalyse different reactions, accept a wide range of substrates, and remain active in different media (e.g. aqueous, organic, ionic liquids, supercritical fluids) and over a wide temperature and pH range. These attributes, along with their successful immobilisation and bioengineering improvements, favour their compatibility with numerous applications [4–6].

Lipase-catalysed [trans]esterification and chiral resolution of carboxylic, terpenoid, and phenyl alcohols are some of the most important processes of interest for the F&F, food, pharmaceutical, and personal care industries. Similarly, the esterification of fatty acids is in demand in the same fields. Esters generally exhibit strong and distinctive aromas, lower polarity, lower boiling point (higher volatility), and fewer skin-sensitising properties than their respective alcohols or acids, due to the derivatisation of the hydroxyl group by alkyl groups. These physical and sensory aspects favour their incorporation into different formulations as emulsifiers, thickening agents, emollients, aroma enhancers, fragrances, or flavours.

Following a recent screening and optimisation study [7], the present work focuses on the application of the immobilised *Candida antarctica* lipase A (CALA)—identified as the best-performing commercial biocatalyst in previous stages of this study—to the biotransformation of terpenoid and phenyl alcohols in essential oil samples. Unlike other similar studies targeting only one or a few samples or standards [5, 8–12], the performance of the aforementioned biocatalyst towards an extensive number of alcohol substrates in 35 natural samples of different complexities is investigated here, in order to better understand the selectivity and inhibition effects (if any) involved in such processes. Comprehensive two-dimensional gas chromatography with mass spectrometry detection (GC×GC‒MS) was used for its superior resolution and improved compound identification capacity, enabling the qualitative assessment of reaction changes in samples of different complexities. The comparison between the position of the peaks in the 2D chromatograms of the same sample before and after the enzymatic processing reaction can readily display the absence or reduction in abundance of the alcohols (substrates), and corresponding appearance of the esters (products).

## Experimental

### Chemicals, enzyme, and samples

The list of chemicals used in this study includes vinyl acetate (99%), acetone (99%), *n*-alkanes C_8_-C_26_ (99%), all supplied by Sigma-Aldrich (Castle Hill, NSW, Australia), and *n*-hexane (99%), obtained from Merck (Bayswater, VIC, Australia). The enzyme lipase CALA (*Candida antarctica* A, Batch# BCBV6128) immobilised in Immobead 150 was purchased from Sigma-Aldrich (Castle Hill, NSW, Australia). The commercial essential oils (E.O.) and their sample codes and suppliers are as follows: bergamot (BERG), boronia (BOR), cedarwood atlas (CDWA), cedarwood Virginian (CDWV), cinnamon (CINM), citronella (CIT), clary sage (CLARY), clove (CLO), cypress (CYP), geranium (GER), jasmine (JAS), kanuka (KNK), lavender (LAV), lemongrass (LMG), myrrh (MYR), pine (PINE), peppermint (PPMP), patchouli (PTC), rose absolute (ROSE), rosemary (ROSM), sandalwood Australian (SDWA), sandalwood Indian (SDWI), tea tree (TTO), and vetiver (VTV1) were kindly donated by Australian Botanical Products (Hallam, VIC, Australia). Additionally, copaiba (COP), eucalyptus (EUCR), frankincense (FKI), kaffir lime (KFL), manuka (MNK), masoi bark (MSB), nutmeg (NMG), neroli (NRL), sweet orange (SWORG), ylang-ylang (YY), and another vetiver (VTV0) samples were obtained from other research partners or purchased online from Amazon Australia. More specifications about the samples are provided in the Appendix S1 of the Supplementary Information.

### Lipase enzyme transesterifications

Lipase reactions were performed according to our previous optimisation study [7], in 2 mL vials, at 40 °C, with 350 rpm magnetic stirring, using 3 mg/mL of CALA enzyme product, 100 mg/mL (10% w/v) of the essential oil samples as the substrate, and 1 mL of a vinyl acetate/hexane solution (6:4) as acyl donor/solvent system. Citronella oil was initially used as a general test sample in different concentrations (1, 10, 100, 500, and 900 mg/mL, which is equivalent to 0.1, 1, 10, 50, and 90% w/v). Based on this initial test, a 10% w/v concentration was selected to perform the reactions with all other essential oils. The reactions were terminated after 48 h by decanting the reaction fluid from the immobilised enzyme and transferring the recovered solution to a new vial. The samples were then diluted with hexane (1:10) for the GC×GC‒MS analysis.

### GC×GC‒MS analysis

The chromatographic analyses were performed on an Agilent 7890A GC system, with automatic injector G4513A, coupled with a 5975C single quadrupole mass spectrometer detector (MSD; Agilent Technologies, Mulgrave, Australia), and a J&X SSM1800 solid-state modulator system (J&X Technologies, Nanjing, China). The system was equipped with a DB-5 ms UI (30 m × 0.25 mm × 0.25 µm) as the first dimension (^1^D) column, and a SLB-IL60 (1.5 m × 0.1 mm × 0.1 µm) as the second dimension (^2^D) column. Fused silica capillaries were used as the modulator column (1 m × 0.15 mm) and the transfer line to the MSD (0.45 m × 0.1 mm). All columns were connected by glass press fits.

The chromatographic method was adapted from previous studies [7, 13], in order to achieve a satisfactory separation for most analytes in all the different samples. Injections (0.6 µL of a ~ 10 mg/mL sample solution) were made at 250 °C, in split mode (20:1), with helium (grade 99.99%) as carrier gas, flow 1 mL/min. The oven temperature program commenced at 60 °C (1 min), with heating at 5 °C/min to 150 °C, then at 3 °C/min to 210 °C, and finally at 15 °C/min to 285 °C (1 or 16 min hold time, depending on the sample); a post-run cooling to 60 °C and oven equilibration was set at 12 min. Other settings were as follows: transfer line to the MSD was held at 285 °C, with the MSD set to scan mode, 12,500 u/s speed, 23.5 scans/s, 80 threshold, 50–350 *m/z* range, 4.6 min solvent delay, 70 eV electron ionisation energy, source and quadrupole temperatures at 230 °C and 150 °C, respectively.

The solid-state modulator entry oven was set according to the GC oven, while the exit oven was set to 80 °C (1 min), 5 °C/min to 170 °C, 3 °C/min to 230 °C, 15 °C/min to 305 °C (1 or 16 min hold time, depending on the sample). The modulator’s cold trap was set to 10 °C (3 min), − 50 °C/min to − 50 °C (5 min), 2 °C/min to 20 or 50 °C (0.80 min). The modulation period was 6 s, desorption time was 1 s, and no delay was used. The final hold time for the GC and modulator methods was increased for the essential oil samples containing a high number of sesquiterpenes.

Agilent MassHunter workstation Qualitative Analysis Version 10.0 and J&X Canvas Version W1.5.14.30115 were the main software used for data processing and 2D chromatogram generation, respectively. The chromatographic peaks were integrated and tentatively identified, according to NIST 11 mass spectrometry library match. Alternatively, for the peaks of sesquiterpene alcohols and esters in vetiver that were not found in the NIST library, the similarity with the mass spectrum and retention indices was compared with those reported by Tissandié et al. [14] and Notar Francesco et al. [9]. The van den Dool & Kratz retention indices were calculated through correlation with the analysis of C_8_-C_25_ *n*-alkanes using the same conditions and based on ^1^D column retention (see Appendix S3 of the Supplementary Information for *n*-alkanes data). Although the quantitative analysis was not the aim of this work, the percentage difference between the chromatographic areas of the alcohol peaks before and after the reaction was used to assess the conversion percentage (C%) of the substrates, which was related to the process efficiency.

## Results and discussion

The initial assessment of the bioreaction with citronella oil at different concentrations demonstrated that the total conversion of the major alcohols (citronellol and geraniol) was completed within 24 h for the test solutions containing 0.1% to 10% w/v of the oil. When 50% oil was used, the same analytes had an average conversion of about 95% after 24 h and 100% after 48 h reaction. No significant changes were observed for the 90% w/v solution within 48 h of reaction, suggesting that the enzyme efficiency is much reduced at such a high sample concentration. Thus, a 10% w/v essential oil sample concentration and 48 h reaction time were chosen as the most suitable working condition for further experiments, taking into consideration that there are differences in the composition and analyte abundance in the other samples, such as the presence of secondary and tertiary alcohols, which may take longer to react.

Additionally, these results indicate that the previously optimised reaction conditions [7] are similarly efficient for an essential oil sample at a concentration (mg/mL) around 167 times higher than that of the enzyme, and it may continue to produce the desired reaction products slightly above this level, provided the reaction is left to proceed for a longer duration. This is important information for future scale-up studies, even though the essential oil sample concentrations are not directly equivalent to the concentration of the total substrate alcohols within each sample.

The GC×GC‒MS method enabled the tentative identification of 125 target analytes, including 79 alcohols and 46 esters (Table 1), within the 35 essential oil samples studied, before and after enzyme bioprocessing. The compounds that did not satisfactorily match the reference mass spectrum and retention indices were not included in the table.

**Table 1:** Alcohols and their corresponding esters identified using GC×GC‒MS analysis in the suite of essential oil samples investigated

Peak #	Alcohols	Type	^1^*t*_R_ (min)	^2^*t*_R_ (s)	RI (calc)	RI (lib)	C %		Peak #	Esters	^1^*t*_R_ (min)	^2^*t*_R_ (s)	RI (calc)	RI (lib)	Samples
Monoterpenoids															
1	(*Z*)-linalool oxide (furanoid)	3°	14.07	1.62	1073	1074	-		-	-	-	-	-	-	BERG, CYP, CLARY, LAV, GER
2	sabinene hydrate	3°	14.17	1.26	1076	1070	-		-	-	-	-	-	-	PPMP, FKI, ROSM, CIT, NMG
3	*β*-linalool	3°	14.89	1.35	1101	1099	-		25	linalyl acetate*	19.27	1.20	1252	1257	LAV, CLARY, BERG, NRL, GER
4	fenchol	2°	15.67	1.30	1128	1113	28		26	fenchyl acetate	18.47	1.15	1224	1223	KNK, ROSM, PINE, LAV, CYP
5	trans-p-mentha-2,8-dien-1-ol	3°	15.67	1.51	1128	1123	-		-	-	-	-	-	-	BERG, FKI, LMG, ROSM, SWORG
6	cis-p-mentha-2,8-dien-1-ol	3°	16.07	1.79	1141	1140	-		-	-	-	-	-	-	BERG, FKI, LMG, PINE
7	trans-pinocarveol	2°	16.37	1.57	1152	1139	100		27	trans-pinocarvyl acetate	20.67	1.58	1300	1297	KNK, CYP, FKI, NMG, ROSM
8	*β*-terpineol	3°	16.47	1.54	1155	1153	-		28	*β*-terpinyl acetate*	20.17	1.30	1283	1317	NRL, ROSM, BOR, BERG, EUCR
9	cis-verbenol	2°	16.47	2.01	1155	1142	100		29	cis-verbenyl acetate	20.47	1.55	1293	1279	FKI, PINE, ROSM, KNK, MNK
10	*α*-phellandren-8-ol	3°	16.57	1.66	1159	1167	-		-	-	-	-	-	-	FKI, LAV, CYP, GER, PINE
11	lavandulol	1°	16.77	1.64	1166	1170	100		30	lavandulyl acetate	20.17	1.30	1283	1289	LAV
12	isopulegol	2°	16.87	1.25	1169	1163	49		31	isopulegol acetate	19.87	1.19	1273	1285	PPMP, BERG, CLO, JAS, CIT
13	isoborneol	2°	17.07	1.43	1176	1157	32		32	isobornyl acetate	20.57	1.40	1297	1286	ROSM
14	borneol	2°	17.27	1.66	1183	1166	61		33	borneyl acetate	20.47	1.38	1293	1285	ROSM, PINE, LAV, LMG, FKI
15	menthol	2°	17.37	1.60	1186	1166	87		34	menthyl acetate	20.47	1.27	1293	1295	PPMP, GER, ROSM
16	terpinen-4-ol	3°	17.47	1.34	1190	1182	-		35	4-terpinenyl acetate*	21.57	1.41	1330	1301	NRL, TTO, NMG, LAV, EUCR
17	*α*-terpineol	3°	17.87	1.60	1204	1189	-		36	*α*-terpinyl acetate*	22.17	1.46	1350	1350	EUCR, BOR, NRL, TTO, CLARY
18	myrtenol	1°	17.87	2.04	1204	1213	100		37	myrtenyl acetate	21.47	1.41	1327	1327	FKI, KNK, ROSM, BERG, CYP
19	trans-piperitol	2°	18.27	1.68	1217	1208	100		38	piperitol acetate	20.67	1.26	1300	1303	NMG, TTO, EUCR, FKI, PPMP
20	(*Z*)-carveol	2°	18.47	1.87	1224	1219	96		39	carvyl acetate	21.67	1.56	1333	1336	FKI, BERG, KNK, PPMP, SWORG
21	*β*-citronellol	1°	18.57	1.83	1228	1228	100		40	*β*-citronellyl acetate	22.07	1.35	1347	1354	CIT, GER, BOR
22	nerol	1°	18.57	1.84	1228	1228	100		41	neryl acetate	22.37	1.40	1357	1342	CLARY, LAV, NRL, EUCR, LMG
23	geraniol	1°	19.27	1.97	1252	1255	100		42	geranyl acetate	22.97	1.46	1377	1382	CIT, GER, LMG, NRL, ROSE
24	perillyl alcohol	1°	20.87	2.42	1307	1296	100		43	perillyl acetate	24.77	1.98	1434	1436	BERG, FKI, LMG,PPMP
Sesquiterpenoids															
44	hedycaryol	3°	28.57	2.02	1553	1559	-			-					CIT, SDWA, FKI, YY, VTV1
45	elemol	3°	28.57	2.04	1553	1549	-			-					CIT, VTV1, VTV0, MYR, YY
46	nerolidol	3°	28.77	1.18	1560	1564	-		-	-	-	-	-	-	YY, SDWA, CINM, ROSM, NRL
47	spathulenol	3°	29.67	2.01	1588	1577	-		-	-	-	-	-	-	PTC, KNK, GER, CLARY, FKI
48	globulol	3°	30.07	1.95	1600	1580	-		-	-	-	-	-	-	PTC, TTO, CDWV, YY, VTV1
49	guaiol	3°	30.27	1.76	1606	1596	-		-	-	-	-	-	-	YY, GER, SDWA, COP, MYR
50	viridiflorol	3°	30.37	1.87	1609	1591	-		-	-	-	-	-	-	KNK, COP, CDWV, VTV0, MSB
51	widdrol	3°	30.77	2.05	1621	1610	-		-	-	-	-	-	-	CDWV, VTV0, VTV1
52	cedrol	3°	30.97	1.84	1627	1598	-		-	-	-	-	-	-	CDWV, CDWA, CYP, KNK
53	selin-6-en-4α-ol	3°	31.17	1.80	1634	1636	-		-	-	-	-	-	-	VTV0, VTV1
54	cubenol	3°	31.27	1.70	1636	1642	-		-	-	-	-	-	-	VTV0, VTV1, YY, CDWA, MNK
55	*γ*-eudesmol	3°	31.27	1.85	1636	1631	-		-	-	-	-	-	-	GER, VTV1, VTV0, YY, SDWA
56	*α*-acorenol	3°	31.57	1.51	1646	1649	-		-	-	-	-	-	-	CDWV, SDWA, SDWI, VTV0,VTV1
57	*τ*-cadinol	3°	31.77	1.71	1652	1640	-		-	-	-	-	-	-	FKI, VTV1, YY, MYR, CIT
58	*τ*-muurolol	3°	31.77	1.98	1652	1642	-		-	-	-	-	-	-	YY, VTV0, CIT, MNK, MSB
59	palustrol	3°	31.97	1.30	1658	1568	-		-	-	-	-	-	-	PTC, KNK, TTO, GER, YY
60	*α*-cadinol	3°	32.17	2.09	1664	1653	-		-	-	-	-	-	-	YY, CIT, MSB, COP, CYP
61	*α*-eudesmol	3°	32.27	1.74	1667	1653	-		-	-	-	-	-	-	VTV0, VTV1, SDWA, MSB,COP
62	*β*-eudesmol	3°	32.27	2.06	1667	1649	-		-	-	-	-	-	-	VTV0, VTV1, SDWA, CIT, TTO
63	eudesma-4,11-dien-2-ol	2°	32.28	2.21	1667	1690	98		85	eudesma-4,11-dien-2-ol acetate	36.68	1.87	1803	1830	VTV0, VTV1
64	cyclocopacamphenol	1°	32.47	2.41	1673	1646	100		86	cyclocopacamphenyl acetate	35.58	1.70	1769	1759	VTV0, VTV1
65	*β*-bisabolol	3°	32.58	1.24	1676	1671	-		-	-	-	-	-	-	VTV0, VTV1
66	*α*-santalol	1°	32.77	2.18	1682	1681	92		87	*α*-santalyl acetate	35.97	1.74	1781	1773	SDWA, CDWV
67	patchouli alcohol	3°	32.97	2.13	1688	1660	-		-	-	-	-	-	-	PTC, BOR
68	*α*-bisabolol	3°	33.07	1.46	1691	1684	-		-	-	-	-	-	-	SDWA, CDWA, VTV1, COP, YY
69	ziza-6(13)-en-3-α-ol	2°	33.07	2.74	1691	1677	100		88	ziza-6(13)-en-3-*α*-yl acetate	36.68	1.87	1803	1775	VTV0, VTV1
70	(*Z,Z*)-farnesol	1°	33.09	1.24	1692	1713	100		89	(*Z*,*Z*)-farnesyl acetate	36.78	0.74	1807	1817	SDWA, SDWI, YY, CIT
71	(*Z*)-α-bergamotol	1°	33.17	2.18	1694	1700	99		90	(*Z*)-*α*-bergamotol acetate	36.37	1.45	1794	1790	SDWI, SDWA, CDWV
72	khusian-2-ol	2°	33.57	2.22	1706	1694	-		91	khusian-2-yl acetate	36.28	1.83	1791	1761	VTV0, VTV1
73	juniper camphor	3°	33.67	1.82	1710	1692	-		-	-	-	-	-	-	VTV0, VTV1, COP, MSB, GER
74	*β*-costol	1°	33.87	2.59	1716	1778	100		92	*β*-costol acetate	39.18	1.78	1882	1882	VTV0, VTV1
75	(*E,E*)-farnesol	1°	33.89	1.28	1716	1722	100		93	(*E*,*E*)-farnesyl acetate	37.58	0.76	1832	1843	SDWA, SDWI, VTV0, VTV1
76	isokhusimol	1°	34.17	2.51	1725	-	97		94	isokhusimyl acetate	37.78	1.88	1838	1843	VTV0, VTV1
77	*β*-santalol	1°	34.27	2.21	1728	1715	91		95	*β*-santalyl acetate	37.17	1.45	1819	1813	SDWA, SDWI
78	(*E*)-nuciferol	1°	34.37	3.44	1732	1766	95		96	(*E*)-nuciferyl acetate	37.47	2.02	1828	1837	SDWA, SDWI
79	vetiselinenol	1°	34.47	2.42	1735	1723	100		97	vetiselinenyl acetate	37.98	1.82	1844	1852	VTV0, VTV1
80	(*Z*)-*β*-curcumen-12-ol	1°	35.27	2.27	1760	1746	100		98	(*Z*)-*β*-curcumen-12-yl acetate	37.47	1.74	1828	-	SDWA, SDWI
81	valerenol	1°	35.07	2.63	1753	1736	100		99	valerenyl acetate	39.48	1.44	1891	1832	VTV0, VTV1
82	cis-lanceol	1°	35.47	2.19	1766	1763	100		100	cis-lanceol acetate	38.57	1.58	1863	1860	SDWA, SDWI, PTC
83	khusimol	1°	35.37	2.94	1763	1740	97		101	khusimyl acetate	38.68	2.15	1866	1875	VTV0, VTV1
84	(*E*)-isovalencenol	1°	36.57	2.44	1800	1788	91		102	(*E*)-isovalencenyl acetate	39.78	1.36	1901	1906	VTV0, VTV1
Other alcohols and phenols															
103	1-octen-3-ol	2°	11.27	1.57	979	980	100		117	1-octen-3-yl acetate	14.97	1.01	1104	1111	LAV, PPMP, ROSM, PTC, GER
104	3-octanol	2°	11.77	1.25	997	994	100		118	3-octyl acetate	15.37	1.04	1117	1123	LAV, PPMP, ROSM
105	benzyl alcohol	1°	12.99	5.08	1037	1036	100		119	benzyl acetate	16.78	1.85	1166	1164	JAS, ROSE, BOR, YY, BERG
106	1-octanol	1°	13.97	1.76	1070	1071	100		120	1-octyl acetate	17.97	1.18	1207	1210	SWORG, PPMP, YY
107	phenylethyl alcohol	1°	15.37	4.94	1118	1116	99		121	phenylethyl acetate	19.47	2.40	1259	1258	ROSE, BOR, CINM, GER, NRL
108	*p*-cymen-8-ol	3°	17.57	2.39	1193	1183	-		-	-	-	-	-	-	FKI, ROSM, NMG, BERG, KNK
109	1-decanol	1°	19.87	1.52	1273	1273	100		122	1-decyl acetate	23.87	1.19	1406	1408	FKI, CLARY, PPMP, TTO, ROSE
110	thymol	2°	20.37	3.94	1290	1291	-		-	-	-	-	-	-	CYP, FKI, TTO, ROSM, CDWV
111	cuminol	1°	20.57	3.78	1298	1289	100		123	cuminyl acetate	24.37	2.46	1422	1428	FKI, CYP, LAV, BERG, PINE
112	cinnamyl alcohol	1°	21.09	1.05	1314	1312	100		124	cinnamyl acetate	25.18	3.83	1447	1446	CINM
113	eugenol	2°	22.39	3.25	1357	1357	-		-	-	-	-	-	-	CLO, CINM, CIT, ROSE, JAS
114	isoeugenol	2°	25.29	5.48	1451	1450	-		-	-	-	-	-	-	CINM, VTV1, NMG, CIT, VTV0
115	isophytol	3°	40.75	1.94	1921	1948	-		-	-	-	-	-	-	JAS
116	phytol	1°	43.35	1.87	2107	2114	99		125	phytol acetate	44.56	1.75	2209	2218	JAS

*Esters of tertiary alcohols that were identified in the original samples, not as a product of the enzyme processing

Alcohol types: primary (1°), secondary (2°), and tertiary (3°)

^1^*t*_R_ and ^2^*t*_R_ are the retention times of analytes for the ^1^D and ^2^D columns, respectively

RI (calc) and RI (lib) are the calculated and reference retention indices

C% is the highest percentage conversion observed for each of the alcohols across all the samples (details in the “Experimental” section)

Due to space constraints, only up to 5 examples of samples containing each compound have been included in the table. Sample codes according to “Chemicals, enzyme, and samples”

All compounds in the table were tentatively identified by their correlation with reference mass spectra and retention indices

Thus, the occurrence of a given substrate alcohol in multiple samples was frequently observed, which means that many of these specific analytes were tested multiple times within samples of different compositions and complexities. In general, a consistent efficiency was observed for the conversion of primary alcohols identified in multiple samples, such as citronellol and geraniol, indicating that neither the number of analytes, their chemical diversity, nor their abundance in the samples seems to adversely affect or inhibit the enzyme activity towards these substrates.

**Fig. 1:**
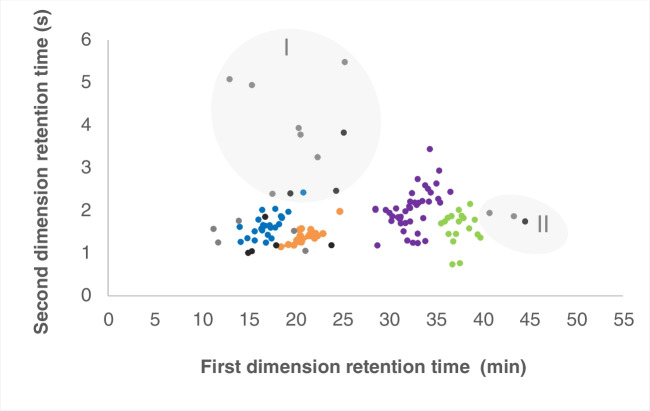
Scatter diagram of the GC×GC‒MS results, demonstrating the relative position of the target analyte peaks in the 2D space (^1^*t*_R_ and ^2^*t*_R_ according to Table 1) and clustering of groups of monoterpene alcohols (blue), monoterpene esters (orange), sesquiterpene alcohols (purple), and sequiterpene esters (green). Other alcohols (grey) and esters (black) are also included, with clustering of groups of phenyl (area I) and diterpenoid (area II) compounds highlighted. GC×GC‒MS analysis was performed using a DB-5/SLB-IL60 (non-polar/polar) column set (see details in the “Experimental” section); hence, generally more polar analytes elute at later retention times in the second dimension

A scatter diagram of the GC×GC‒MS chromatogram (Fig. 1) illustrates the retention time coordinates of the target analytes (i.e. alcohols and esters) and demonstrates the superior separation capacity through additional separation on the ^2^D column, and easy visual assessment of chemical changes that this technique offers. Generally, the relative position of a given analyte peak in the 2D space does not significantly vary from one sample to another, provided that the analytical conditions are kept the same for all the sample sets. This attribute allows an analyst to instantly locate the target analytes across the sample set once they are identified in a sample, to recognise clustering groups with similar chemical class and/or molecular features (e.g. monoterpene alcohols and monoterpene esters), and to determine any differences in the overall chemical profile of the samples, such as the changes resulting from the enzymatic process.

**Fig. 2:**
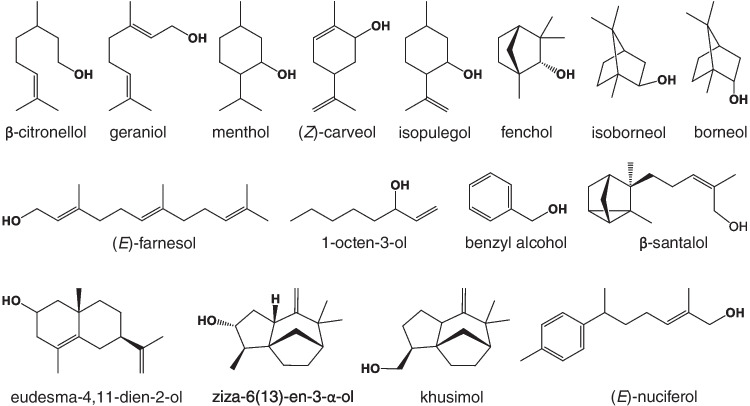
Chemical structures of some of the alcohols esterified by CALA lipase

A total of 42 out of the 79 alcohols identified were successfully converted into their respective acyl esters. The lipase-catalysed esterification reactions were generally quite efficient for primary and secondary alcohols, such as β-citronellol, geraniol, menthol, (*Z*)-carveol, β-santalol, (*E*)-farnesol, (*E*)-nuciferol, 1-octen-3-ol, and benzyl alcohol (Fig. 2), with conversions (C%) of 80 to 100% within 48 h reaction time. Some secondary alcohols, such as fenchol, isopulegol, isoborneol, and borneol (Fig. 2), were only partially converted (C% = 30–60) within the same time. No significant conversion was observed in the tested conditions for tertiary alcohols and phenols, which are major components in many of the samples investigated (e.g. linalool in lavender and eugenol in clove). Despite that, for completeness, the GC×GC‒MS chromatograms of all the tested E.O. have been included in the Appendix S2 of the Supplementary Information.

As demonstrated in our previous study [7], large conversions (C% ≥ 90) can be quickly achieved (within ~ 1 h) for primary alcohol reaction with CALA enzyme, in the tested conditions. Secondary alcohols had around 20% conversion within the same time and around 70% within 24 h in the same study. Thus, in the present work, the reaction was allowed to proceed for 48 h to obtain the highest possible C% for all types of alcohol substrates across all the samples tested. This also seems to have contributed to less variations for most part of the compounds with high C% and identified in multiple samples, such as (*Z*)-carveol (C% = 90–96), as well as citronellol and geraniol which were 100% converted in all of the samples containing them. The highest variations were observed for a few compounds, such as isopulegol (C% = 29–49) and borneol (C% = 34–61). However, it is important to reiterate that these C% are estimated from the difference in the indicative chromatographic areas of these alcohols before and after the reaction, but this was not a quantitative measurement, which was not the aim of this study.

By comparison of all the E.O. investigated, the most significant bioprocessing changes were observed for vetiver and sandalwood, which contain a large number of sesquiterpene alcohols that were successfully esterified. GC×GC‒MS enabled the tentative identification of 18 of those alcohols and their respective esters, as well as other alcohols that were not esterified or are from other groups. The samples showing fewer changes contained lower amounts (in concentration and number) of the enzyme’s preferred substrates (i.e. primary and secondary alcohols).

**Fig. 3:**
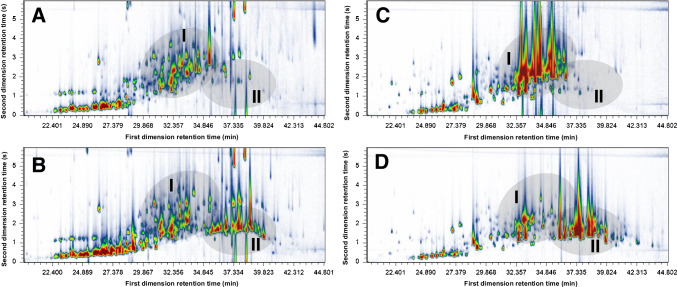
GC×GC‒MS chromatograms of vetiver (**A** and **B**; sample VTV1) and sandalwood (**C** and **D**; sample SDWA) essential oils, illustrating the chemical changes achieved after the lipase-catalysed esterification processing (**B** and **D**), in comparison with the original samples (**A** and **C**). The highlighted areas I and II represent the location in the 2D space where most of the alcohols and esters are found, respectively

The comparison of the 2D chromatograms of the original and bioprocessed vetiver (Fig. 3A and B, respectively) and sandalwood (Fig. 3C and D) samples allows the location of clustering areas in the 2D space corresponding to alcohols (Fig. 3, area I) and esters (Fig. 3, area II), and to clearly observe the chemical changes in the samples obtained with enzymatic processing of the original oil.

Sandalwood samples had conversions of 92% and 91% for the major alcohols α- and β-santalol (Fig. 2) within 48 h, respectively. Other sesquiterpene alcohols, such as (*E*)-farnesol and (*E*)-nuciferol (Fig. 2), were also present in high abundance and were efficiently esterified (90–100%). A number of high-abundance compounds can be observed in sandalwood chromatograms, indicated by large peaks in the contour plot, with some apparent extended tailing. However, to adequately study the effects of lower abundance peaks, some overloading of high-abundance peaks is required (as opposed to diluting the sample).

Specifically for vetiver samples, the enzymatic esterification of secondary terpene alcohols, such as eudesma-4,11-dien-2-ol, and ziza-6(13)-en-3-α-ol, as well as the full conversion of the major alcohol khusimol (Fig. 2), outperforms the biocatalysis results reported by Notar Francesco et al. [9], being closely comparable to their observations for chemical esterification of vetiver alcohols. According to these authors, the acetylation processing can introduce grapefruit, sandalwood, and cedarwood undertones to vetiver oil, as opposed to the usual earthy notes, which can increase the value of this fine fragrance ingredient to almost double the price of the original oil [9].

The use of such chemically diverse natural samples as lipase substrates, aligned with the resolution power of GC×GC‒MS analysis, has enabled the assessment of the enzyme’s performance towards specific compounds, such as the aforementioned vetiver alcohols, for which the pure standards are very expensive or not commercially available.

It is important to highlight that a positive overall odour change arising from the esterification of such complex samples could be related to either the pleasant aroma of the new esters produced, or the suppression of the off-flavour character of the initial alcohols. A lower odour activity of these new esters in comparison to their respective alcohols could also bring other types of odorants present in the original oil to the spotlight as the new key aroma impact compounds in the processed sample. In the case of vetiver, for example, there are different compounds (unrelated to the esterification process) with grapefruit notes present in the raw oil, such as nootkatone, β-vetivone, and valencene, which could also play a more significant role to the overall aroma of the sample after processing. In fact, Tissandié et al. [14] observed in the olfactory assessment of the esterified vetiver oil that the main esters produced were generally odourless or with low odour impact to the overall sample, except for 12-norziza-6(13)-en-2α-yl acetate, which apparently has a stronger vetiver-like note. Thus, the accurate aroma assessment of the overall esterified samples and the identification of their key odorants are complex tasks that require additional analytical steps, which are not covered in the present study.

## Conclusion

The biocatalysed transesterification of a large set of alcohol substrates in 35 E.O. samples was successfully performed in the present study by applying a pre-optimised enzyme reaction with a selected immobilised lipase (CALA). Remarkable results were achieved with the production of esters from primary and secondary alcohols, surpassing previous investigations.

The immobilised CALA enzyme is fully compatible with non-aqueous systems and E.O. samples with different levels of complexity (i.e. number, concentration, and chemical diversity of components), as well as being robust, efficient, easy to apply, and readily removed from the reaction media. Our results also indicate that the scalability of the method should be relatively facile, since a considerable increase in the substrate’s concentration did not compromise the efficiency of the process. Although the present study has not investigated the reusability of this specific catalyst, studies elsewhere have found that other immobilised lipase products can be reused multiple times or in continuous flow systems without significant loss in activity [5, 9].

A panel sensory assessment was originally planned, in order to draw specific conclusions about the odour changes in the samples. However, this is a laborious task that requires a certain number of volunteers and training, which was not possible to do, due to COVID-19 and other issues. For this reason, a more in-depth discussion on the odour changes of the samples was not included in the present study. The method developed here aimed to demonstrate that the molecular changes accompanying enzyme treatment could be followed by using GC×GC analysis, with appropriate conversion of chemical classes. A more complete study with odour assessment is a future objective.

The combination of low-cost natural raw materials, such as some essential oils, with the selectivity, efficiency, and robustness of more environmentally friendly synthetic processes, such as biocatalysis, offers a benign avenue to manufacture novel flavour and fragrance products with improved properties and safety, without compromising the branding associated with “natural” labelling.

As a tool for the discovery of specific composition changes in complex samples, GC×GC‒MS has proven to be a promising technique with superior resolution and identification capabilities, able to generate 2D plots that facilitate the visualisation of the changes achieved with the enzymatic processing, as well as any other differences in the samples’ chemical profiles. The comparison of the GC×GC–MS data of the original oils with their enzymatically transformed products is a straightforward process that generates data with a high information content and outstanding results in comparison with what would be available from a single dimension GC–MS analysis where the target chemical classes are not as readily displayed chromatographically. However, data analysis can still be a laborious and time-consuming task in the absence of efficient, integrated, and user-friendly software. A desirable interface would allow quick, precise, and simultaneous analysis of multiple samples, providing all the main peak parameters and 2D chromatograms (with overlay function) as well as connecting with updated MS databases to provide more accurate compound identification lists.

## Supplementary Information

Below is the link to the electronic supplementary material.

Supplementary file1 (DOCX 11708 KB)
