# Supplementary material for: Lipase-catalysed changes in essential oils revealed by comprehensive two-dimensional gas chromatography
Source: Anal Bioanal Chem. 2023 May 15;415(16):3189–99. doi: 10.1007/s00216-023-04729-0 (PMC10184066; doi:10.1007/s00216-023-04729-0)
Supplement: Supplementary file 1 — Supplementary file1 (DOCX 11708 KB) [file 216_2023_4729_MOESM1_ESM.docx]

**Supplementary Information**

**Lipase-catalysed changes in essential oils revealed by comprehensive two-dimensional gas chromatography**

**Michelle S.S. Amaral, Milton T.W. Hearn and Philip J. Marriott***

Australian Centre for Research on Separation Science, School of Chemistry, Monash University.

Wellington Road, Clayton, VIC 3800, Australia.

*philip.marriott@monash.edu

Analytical and Bioanalytical Chemistry

**Table of contents**

| **Appendix S1.** Essential oil samples table……………………………………………………….………….. | 2 |
| --- | --- |
| **Appendix S2.** GC×GC‒MS chromatograms for the essential oil samples………………….. | 3 |
| **Appendix S3.** GC×GC‒MS chromatogram and table for the *n*-alkanes (C_8_-C_25_)…………… | 11 |
|  |  |
|  |  |
|  |  |

**Appendix S1.** Essential oil samples table

| **Code** | **Essential oil** | **Scientific name** | **Plant part** | **Manufacturer / Supplier** |
| --- | --- | --- | --- | --- |
| BERG | Bergamot | *Citrus bergamia* | Fruit peel | Auroma / ABP |
| BOR | Boronia | *Boronia megastigma* | Flower | Auroma / ABP |
| CDWA | Cedarwood Atlas (*Morocco wild*) | *Cedrus atlantica* | Stem | Essential Therapeutics/ ABP |
| CDWV | Cedarwood Virginian | *Juniperus virginiana* | Stem | Auroma / ABP |
| CINM | Cinnamon | *Cinnamomum cassia* | Bark | ABP |
| CIT | Citronella Java | *Cymbopogon winteranus* | Leaves | ABP |
| CLARY | Clary Sage | *Salvia sclarea* | Leaves | Auroma / ABP |
| CLO | Clove | *Syzygium aromaticum* | Flower buds | Auroma / ABP |
| COP | Copaíba | *Copaifera martii* H. | Gum resin | Pacific Scents |
| CYP | Cypress | *Cupressus sempervirens* | Stems/ Leaves | Essential Therapeutics / ABP |
| EUCR | Eucalyptus | *Eucalyptus radiata* | Leaves | Australian Lab |
| FKI | Frankincense | *Boswellia serrata* | Gum resin | Primavera |
| GER | Geranium | *Geranium spp.* | Flower | Essential Therapeutics / ABP |
| JAS | Jasmine | *Jasminum spp.* | Flower | Essential Therapeutics / ABP |
| KFL | Kaffir Lime | *Citrus hystrix* | Leaves | Natoria |
| KNK | Kanuka | *Kunzea ericoides* | Leaves | Essential Therapeutics / ABP |
| LAV | Lavender | *Lavandula angustifolia* | Leaves/ Flower | Auroma / ABP |
| LMG | Lemongrass *(Nepal, Organic)* | *Cymbopogon flexuosus* | Leaves | Essential Therapeutics / ABP |
| MNK | Manuka | *Leptospermum scoparium* | Leaves | Gya labs |
| MSB | Masoi Bark | *Cryptocarya massoy* | Bark | Natoria |
| MYR | Myrrh | *Commiphora myrrha* | Gum resin | Essential Therapeutics / ABP |
| NMG | Nutmeg | *Myristica fragans* | Seeds | Natoria |
| NRL | Neroli | *Citrus aurantium*L. | Flower | Gya labs |
| PINE | Dwarf Pine | *Pinus pumila* | Leaves | ABP |
| PPMP | Peppermint | *Mentha piperita* | Leaves | Essential Therapeutics / ABP |
| PTC | Patchouli *(Indonesia)* | *Pogostemon cablin* | Leaves | Essential Therapeutics / ABP |
| ROSE | Rose absolute | *Rosa damascena* | Flower | Auroma / ABP |
| ROSM | Rosemary | *Rosmarinus officinalis* | Leaves | Essential Therapeutics / ABP |
| SDWA | Sandalwood Australian | *Santalum spicatum* | Root Heartwood | Auroma / ABP |
| SDWI | Sandalwood East Indian | *Santalum album* | Heartwood | ABP |
| SWORG | Sweet Orange | *Citrus sinensis* | Fruit peel | Gya labs |
| TTO | Tea Tree | *Melaleuca alternifolia* | Branch / Leaves | Auroma / ABP |
| VTV0 | Vetiver *(Indonesia)* | *Chrysopogon zizanioides* | Roots | Natoria |
| VTV1 | Vetiver *(Indonesia)* | *Chrysopogon zizanioides* | Roots | Essential Therapeutics / ABP |
| YY | Ylang-Ylang | *Cananga odorata* | Flower | Natoria |

**The sources of the essential oil samples are provided in the manuscript “Experimental” Section 2.1.**

**Appendix S2.** GC×GC‒MS chromatograms for the essential oil samples.

The GC×GC‒MS procedures employed in this study are described in the manuscript Experimental Section 2.3.

The First dimension retention time and Second dimension retention time are abbreviated as ^1^*t*_R_ and ^2^*t*_R_ here.

Some of the main areas in the 2D plots of compounds undergoing changes (that either are reduced in the original oil, or are generated in the product) are highlighted in the chromatograms of the “before enzyme reaction”.

**BEFORE ENZYME REACTION AFTER ENZYME REACTION**

Bergamot (BERG)


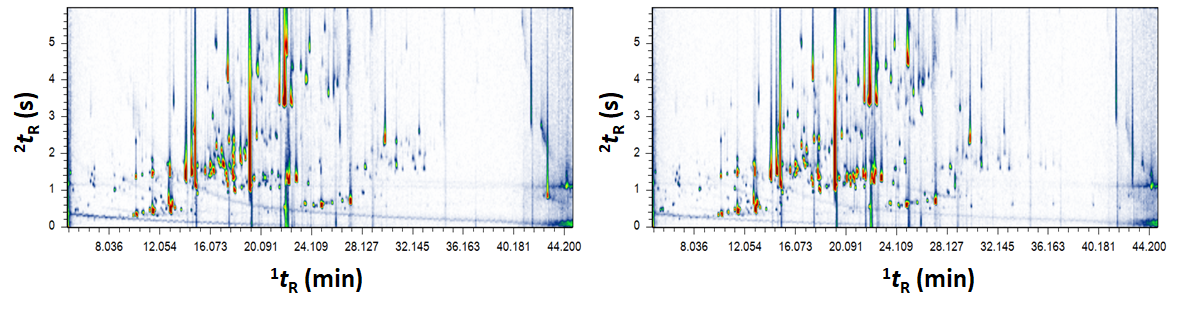


Boronia (BOR)


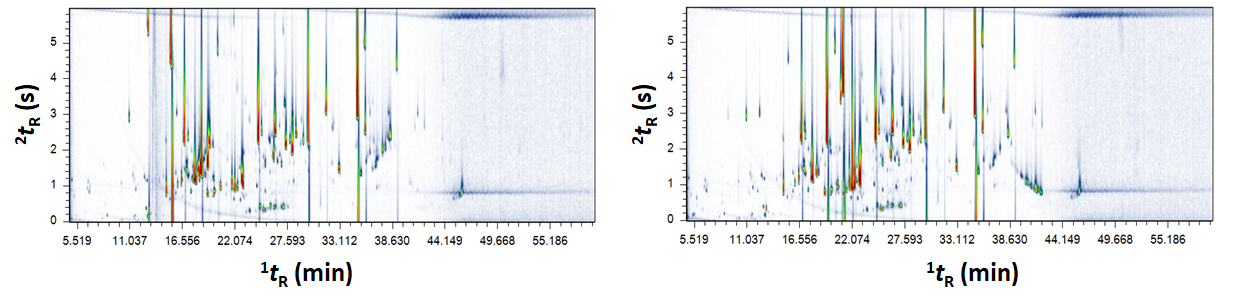


Cedarwood Australian (CDWA)


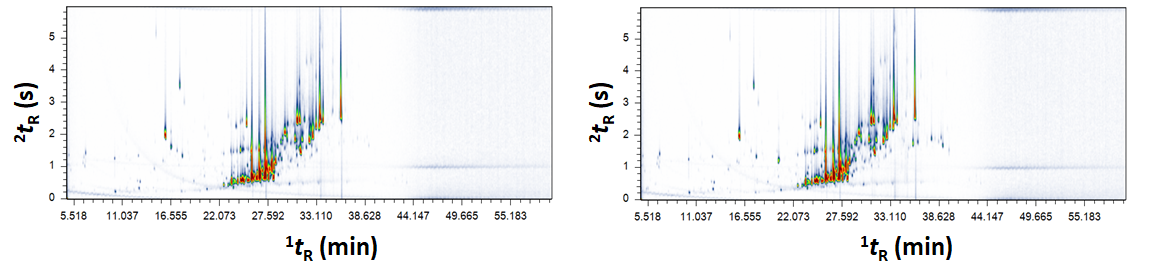


Cedarwood Virginian (CDWV)


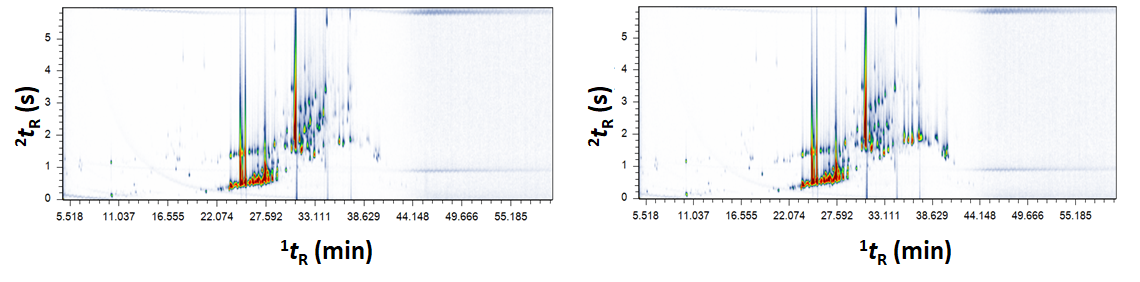


Cinnamon (CINM)


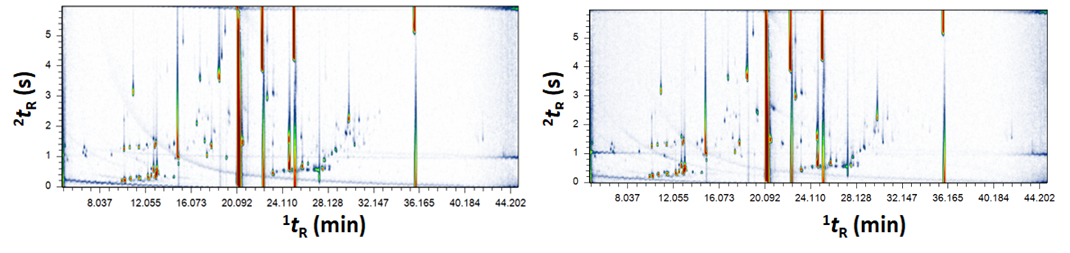


Citronella (CIT)


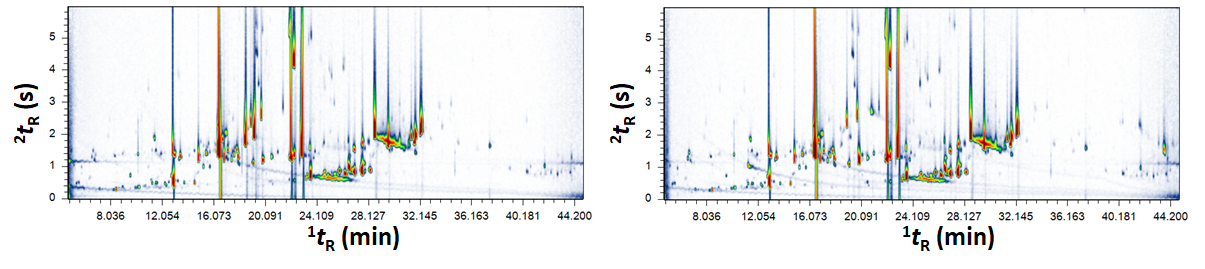


Copaíba (COP)


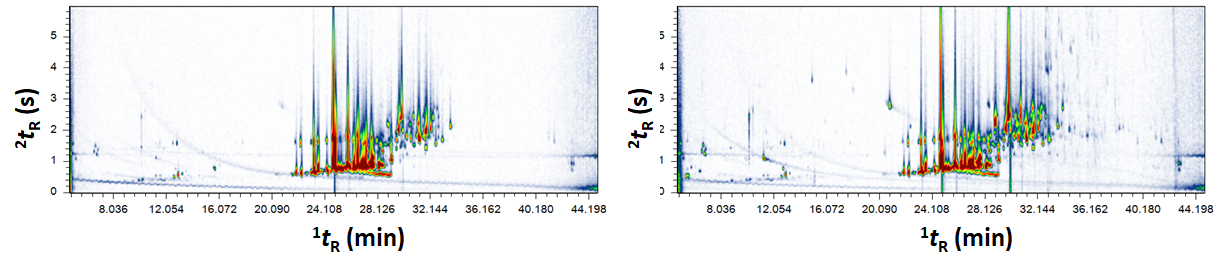


Clary Sage (CLARY)


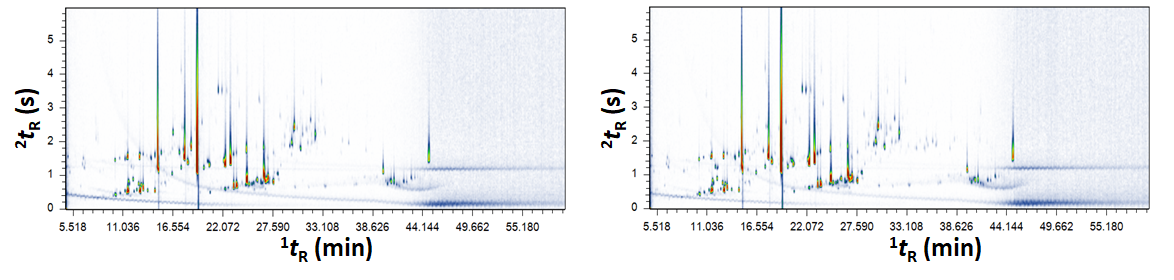


Clove (CLO)


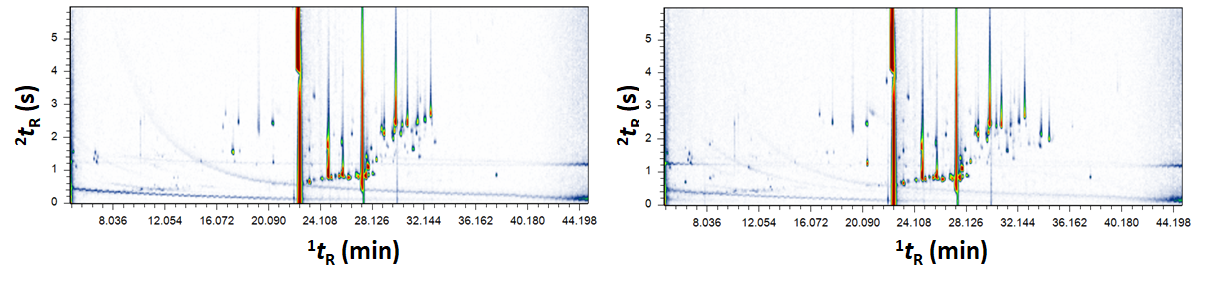


Cypress (CYP)


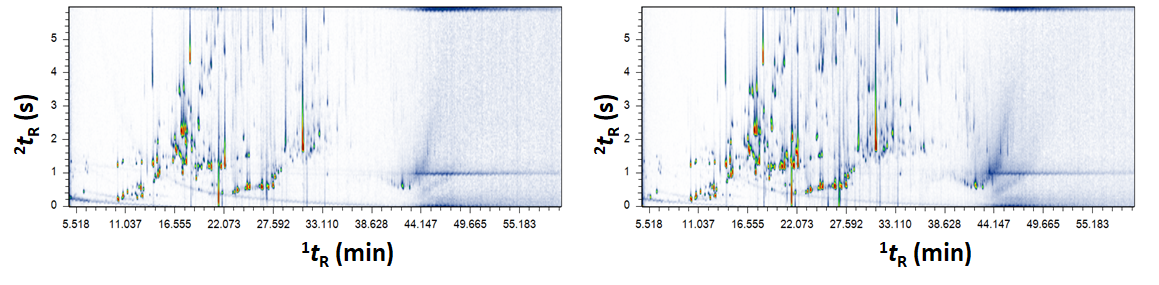


Eucalyptus R. (EUCR)


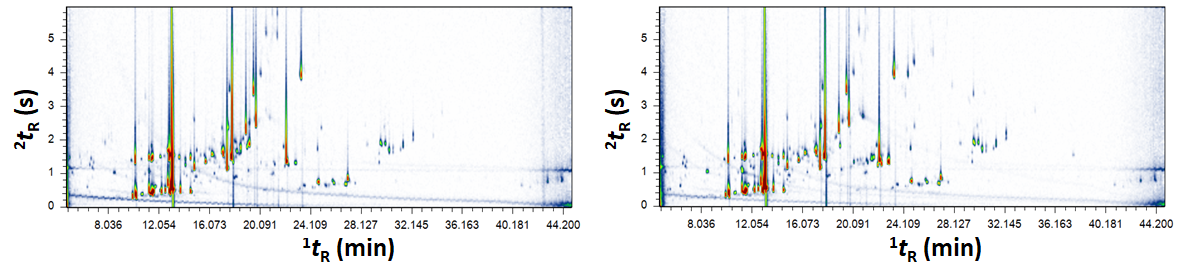


Frankincense (FKI)


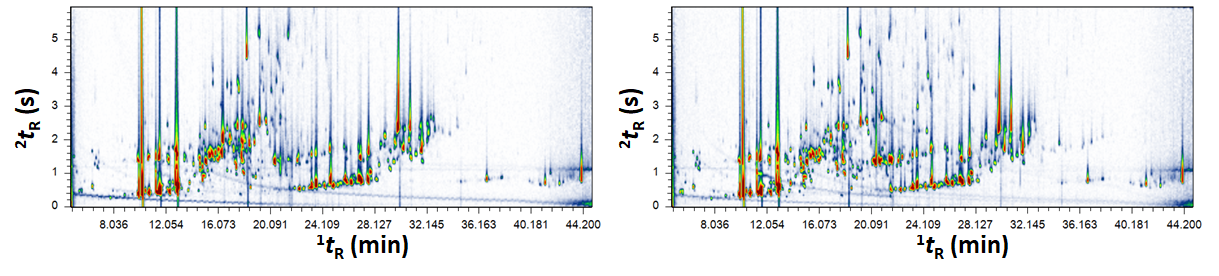


Geranium (GER)


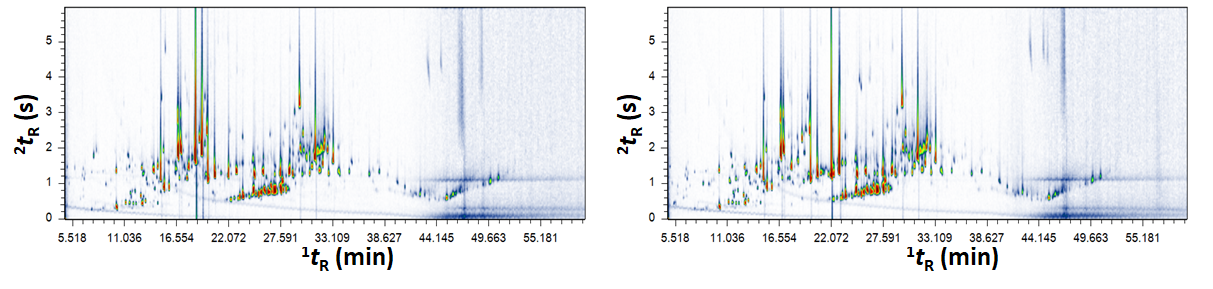


Jasmin (JAS)


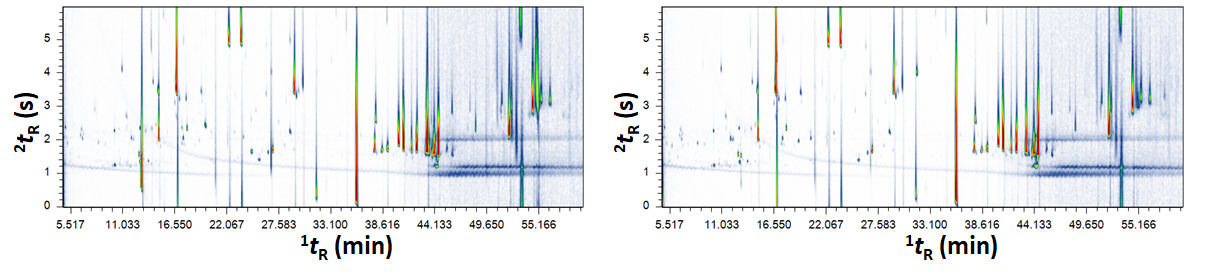


Kaffir Lime (KFL)


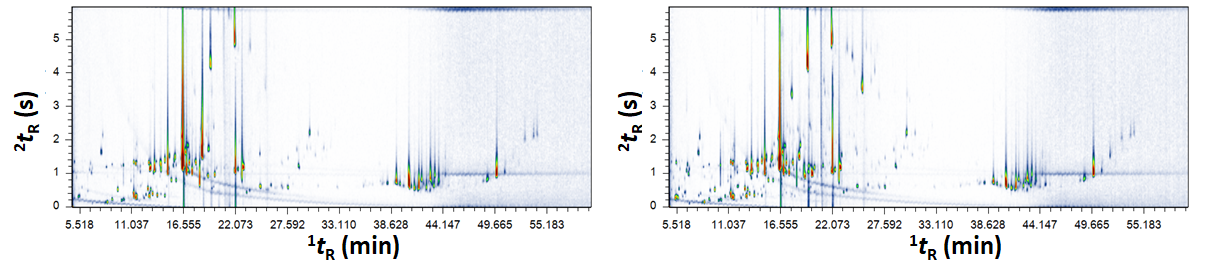


Kanuka (KNK)


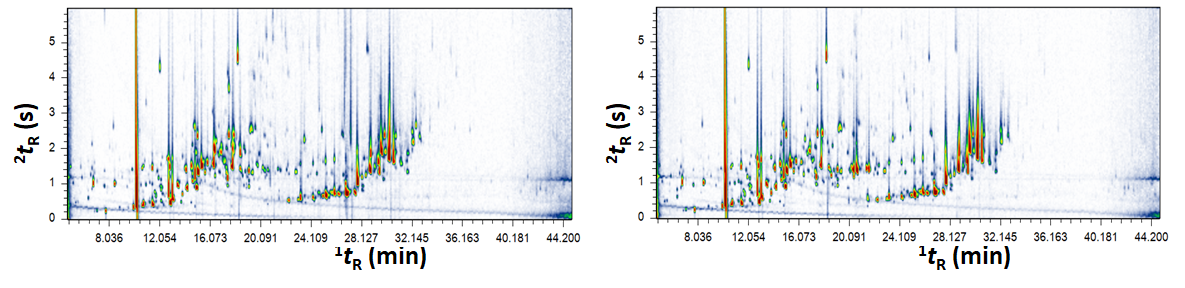


Lavender (LAV)


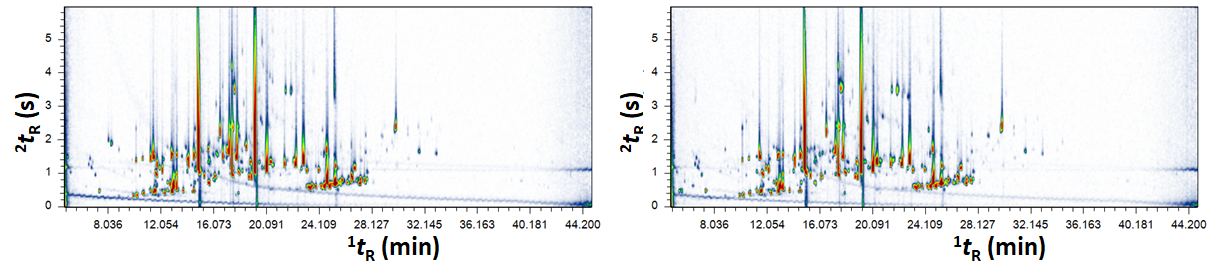


Lemongrass (LMG)


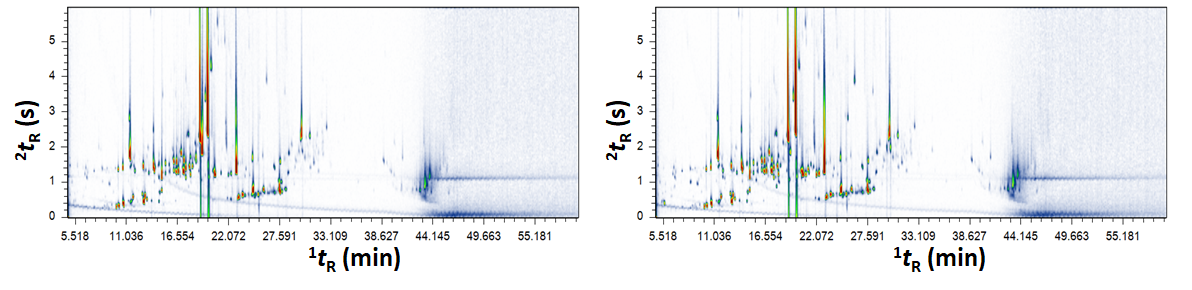


Manuka (MNK)


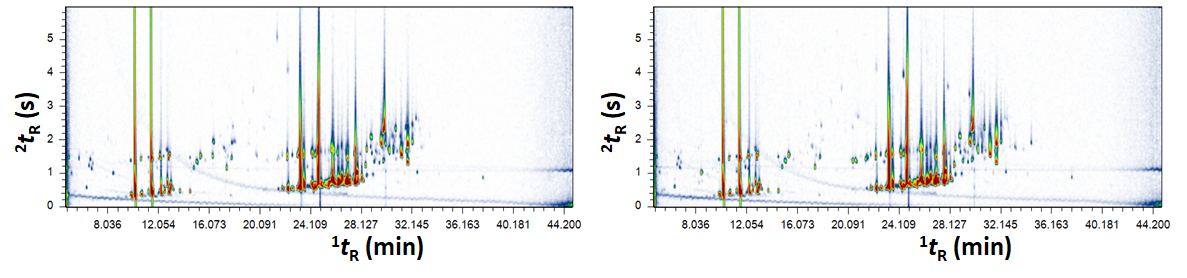


Masoi Bark (MSB)


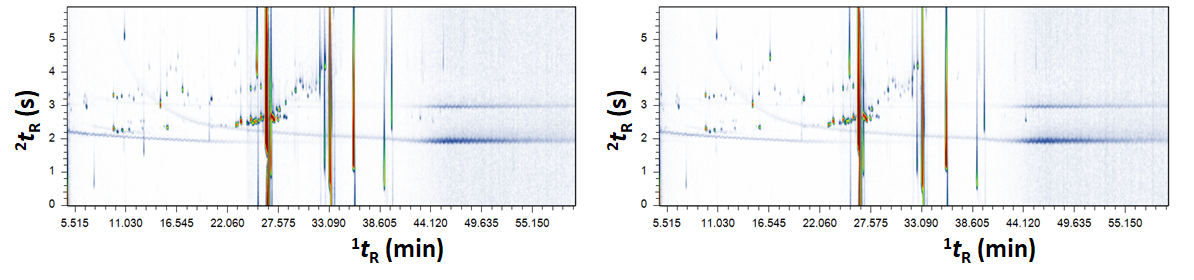


Myrrh (MYR)


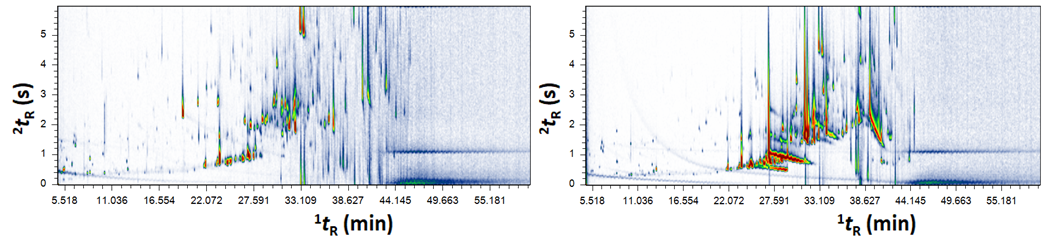


The unusual ‘peaks’ in the form of bands (indicated here by arrows) observed for MYR in the chromatogram “after enzyme reaction” and other samples, such as CIT and COP, are attributed to an interconversion phenomenon that occurs on the column for certain compounds during the GC separation. The tentative identification of the compounds presenting this elution pattern in the present study indicates they overall belong to the class of terpene hydrocarbons, which is unrelated to the enzyme’s targeted substrates (i.e. alcohols or esters).

Neroli (NRL)


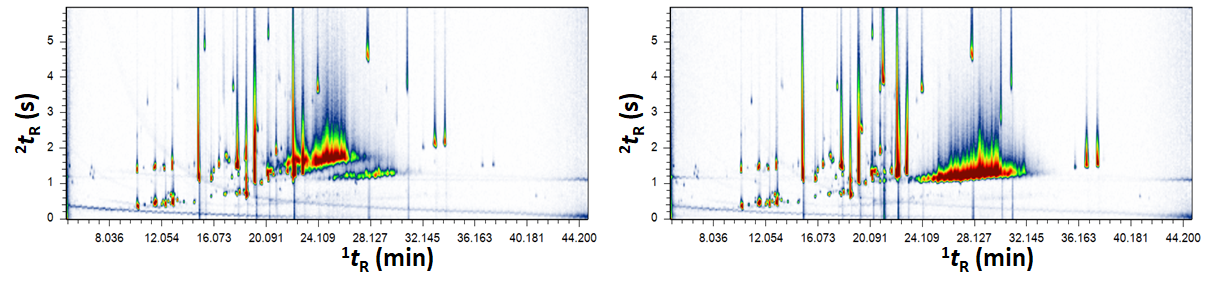


Nutmeg (NMG)


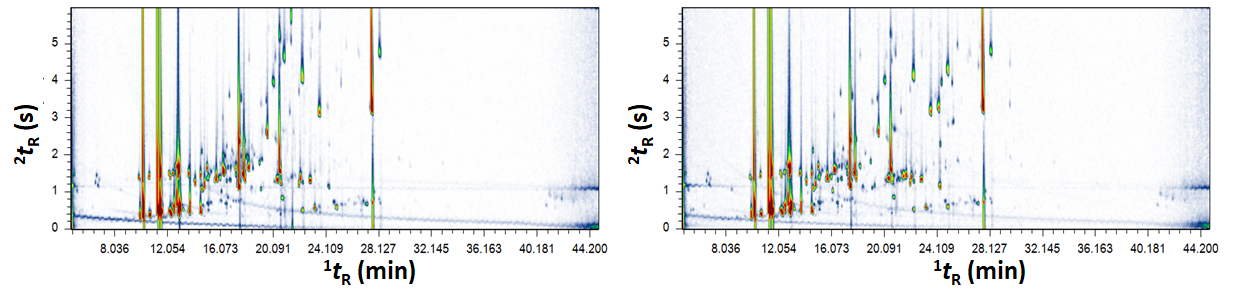


Patchouli (PTC)


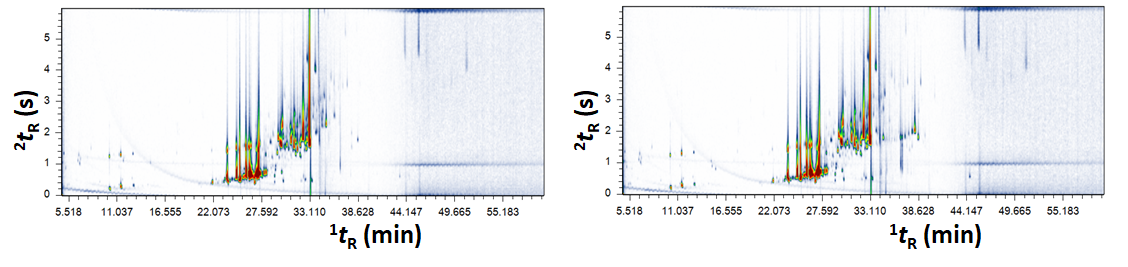


Peppermint (PPMP)


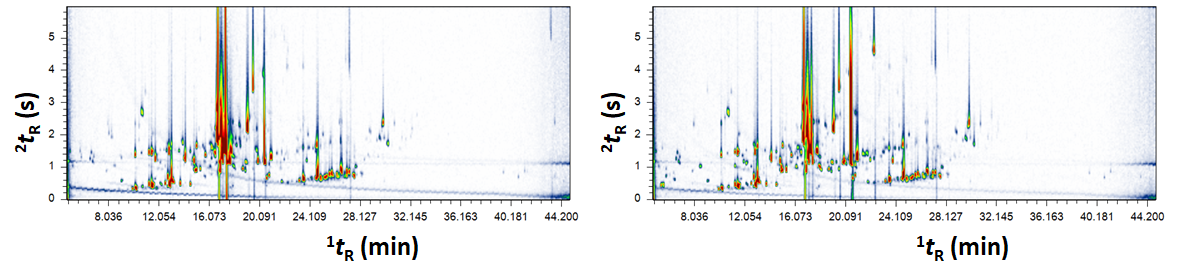


Pine (PINE)


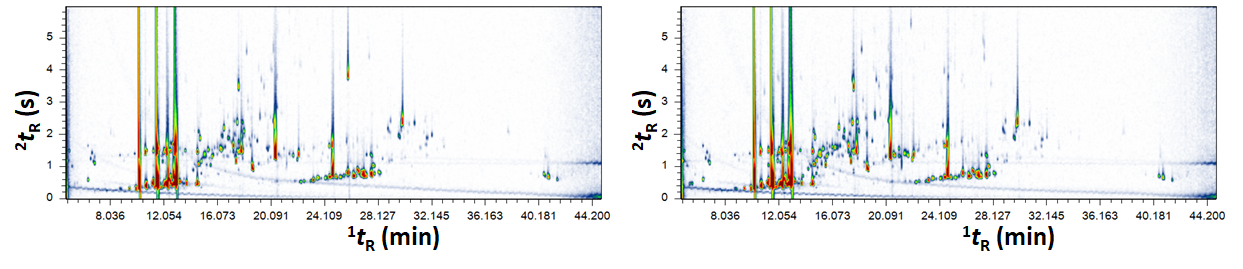


Rose (ROSE)


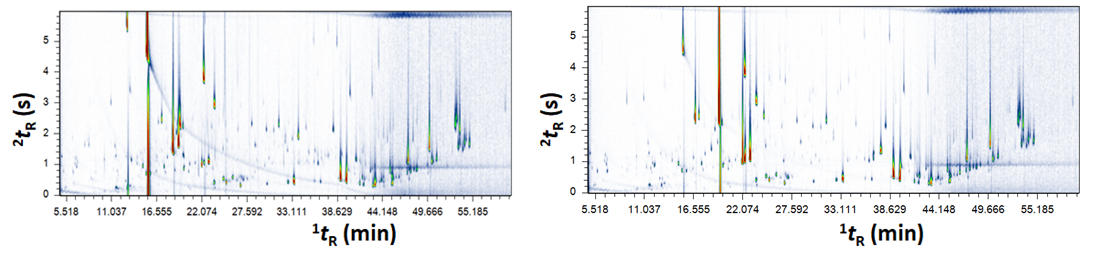


Rosemary (ROSM)


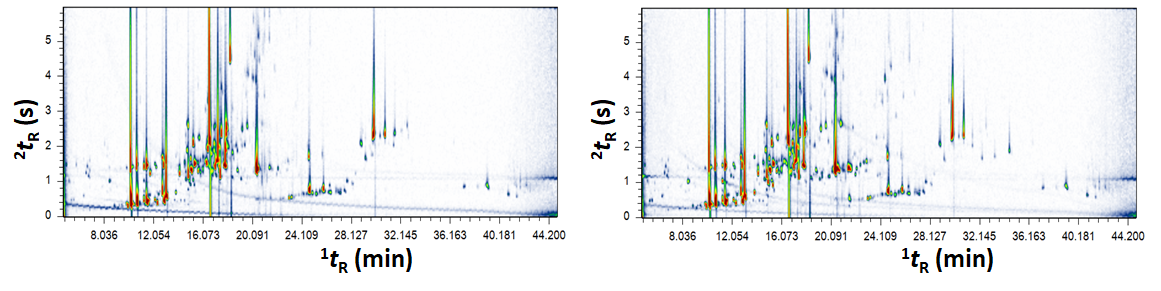


Sandalwood Australian (SDWA)


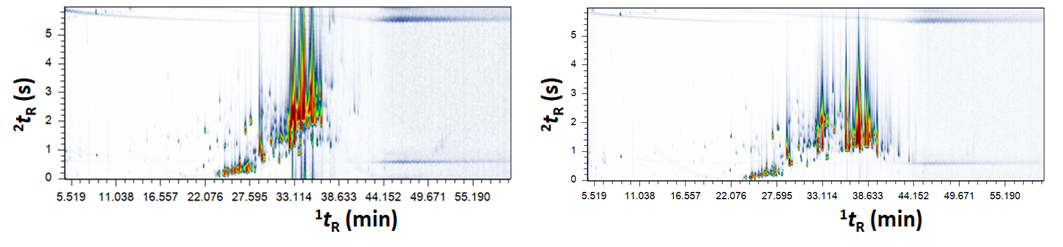


Sandalwood Indian (SDWI)


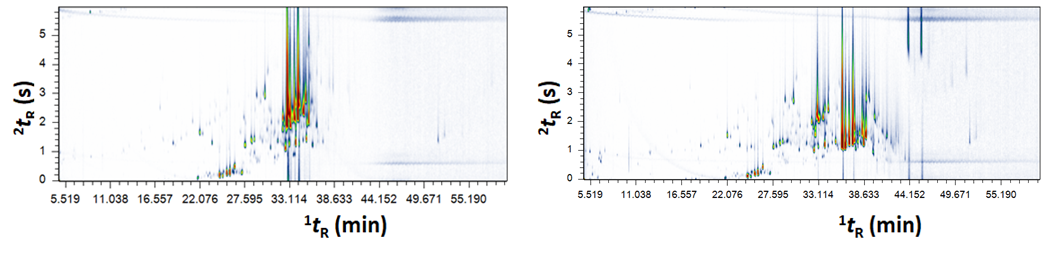


Sweet Orange (SWORG)


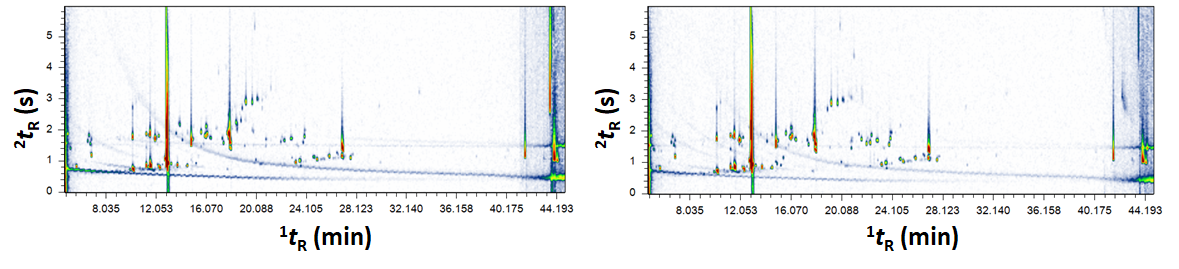


Tea Tree (TTO)


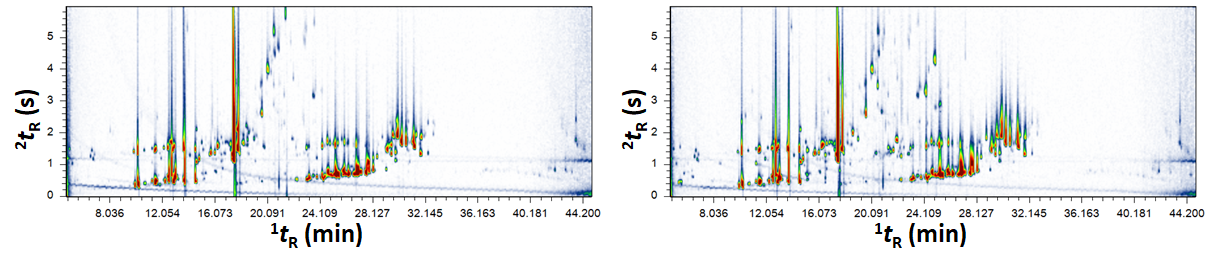


Vetiver (VTV0)


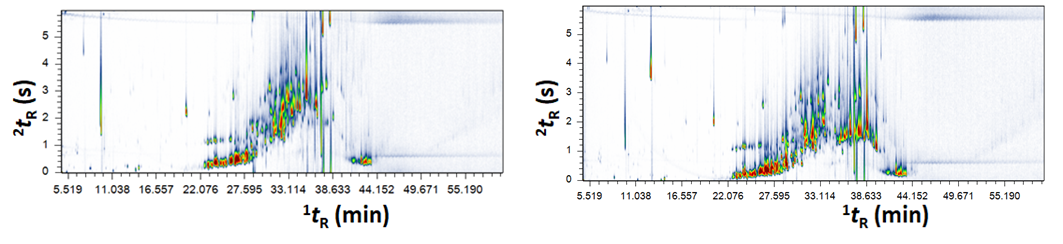


Vetiver (VTV1)


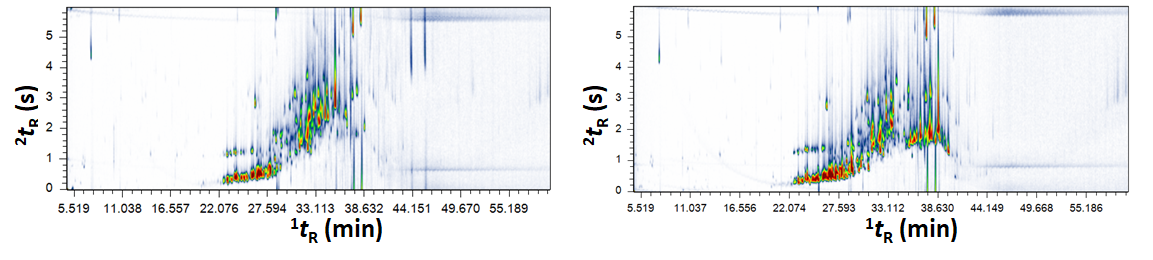


Ylang-Ylang (YY)


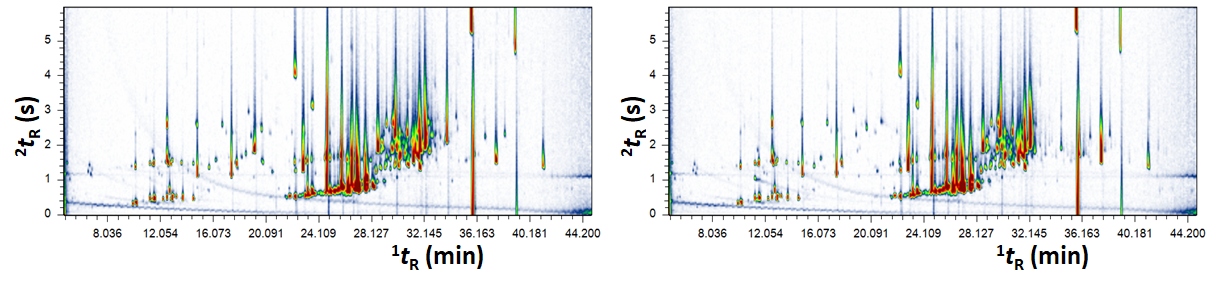


**Appendix S3.** GC×GC‒MS chromatogram and table for the *n*-alkanes (C_8_-C_25_)


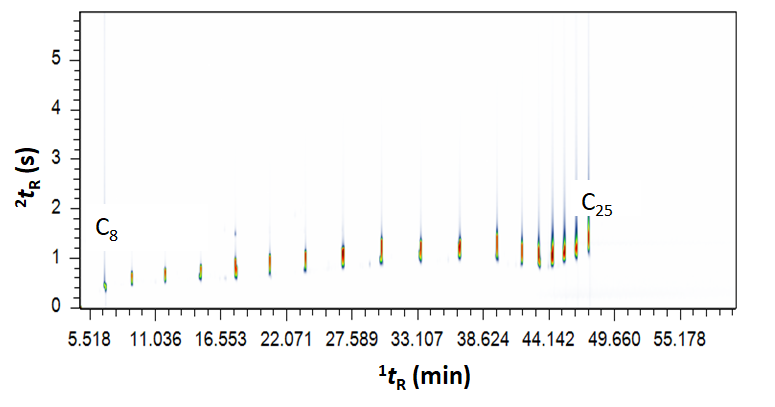


| C*_n_* | ^1^*t*_R_ (min) | ^2^*t*_R_ (s) |
| --- | --- | --- |
| 8 | 6.87 | 0.39 |
| 9 | 9.07 | 0.54 |
| 10 | 11.87 | 0.60 |
| 11 | 14.87 | 0.66 |
| 12 | 17.77 | 0.77 |
| 13 | 20.67 | 0.85 |
| 14 | 23.67 | 0.94 |
| 15 | 26.87 | 1.02 |
| 16 | 30.07 | 1.11 |
| 17 | 33.37 | 1.11 |
| 18 | 36.57 | 1.11 |
| 19 | 39.77 | 1.19 |
| 20 | 41.87 | 0.95 |
| 21 | 43.27 | 0.97 |
| 22 | 44.47 | 0.98 |
| 23 | 45.47 | 1.08 |
| 24 | 46.47 | 1.16 |
| 25 | 47.47 | 1.31 |
